# Natural Products in Therapeutic Management of Multineurodegenerative Disorders by Targeting Autophagy

**DOI:** 10.1155/2021/6347792

**Published:** 2021-09-13

**Authors:** Sibhghatulla Shaikh, Khurshid Ahmad, Syed Sayeed Ahmad, Eun Ju Lee, Jeong Ho Lim, Mirza Masroor Ali Beg, Amit K. Verma, Inho Choi

**Affiliations:** ^1^Department of Medical Biotechnology, Yeungnam University, Gyeongsan 38541, Republic of Korea; ^2^Research Institute of Cell Culture, Yeungnam University, Gyeongsan 38541, Republic of Korea; ^3^Faculty of Medicine, Ala-Too International University, Bishkek, Kyrgyzstan; ^4^Department of Biotechnology, Jamia Millia Islamia, New Delhi, India

## Abstract

Autophagy is an essential cellular process that involves the transport of cytoplasmic content in double-membraned vesicles to lysosomes for degradation. Neurons do not undergo cytokinesis, and thus, the cell division process cannot reduce levels of unnecessary proteins. The primary cause of neurodegenerative disorders (NDs) is the abnormal deposition of proteins inside neuronal cells, and this could be averted by autophagic degradation. Thus, autophagy is an important consideration when considering means of developing treatments for NDs. Various pharmacological studies have reported that the active components in herbal medicines exhibit therapeutic benefits in NDs, for example, by inhibiting cholinesterase activity and modulating amyloid beta levels, and *α*-synuclein metabolism. A variety of bioactive constituents from medicinal plants are viewed as promising autophagy controllers and are revealed to recover the NDs by targeting the autophagic pathway. In the present review, we discuss the role of autophagy in the therapeutic management of several NDs. The molecular process responsible for autophagy and its importance in various NDs and the beneficial effects of medicinal plants in NDs by targeting autophagy are also discussed.

## 1. Introduction

Autophagy is a fundamental, exceptionally well-controlled process in the eukaryotic cell recycling system under different states of cellular stress. Autophagy plays an essential role in cell survival and maintenance by degrading cytoplasmic organelles, macromolecules, and misfolded proteins and, thus, facilitates the recycling of cellular content as breakdown products [1, 2]. The literal meaning of autophagy is “self-eating,” and this process involves the intracellular capture of unnecessary proteins, lipids, and organelles and directing them for their further degradation in the lysosomal compartment [3]. Thus, autophagy performs a vital role in the overall homeostasis of proteins and, eventually, cells and retains metabolic balance between the synthesis and degradation of cytoplasmic materials and their subsequent reprocessing. Thus, autophagy is a natural cellular mechanism that sustains cellular homeostasis under various conditions [4]. Many other functions of autophagy have been identified in the presence of pathological processes such as infectious diseases, myocardial diseases, diabetes, neurodegenerative diseases (NDs), and various cancers [5, 6].

There are typically three types of autophagy: macroautophagy, microautophagy, and chaperone-mediated autophagy (CMA), all of which have specific roles that depend on the cellular microenvironment, signals, and organs, but all usually involve proteolytic degradation of cytosolic components inside lysosomes [7]. Macroautophagy is the most important degradation pathway and involves the formation of double-membrane vesicles called autophagosomes in cytoplasm. In fact, the term “autophagy” typically refers to this process, unless otherwise specified. Macroautophagy may further be classified as mitophagy, nucleophagy, pexophagy, aggrephagy, and xenophagy [8]. Pathological studies have shown that macroautophagy deficiencies may be involved in the development of NDs [9]. In microautophagy, cellular constituents are taken up by lysosomes by direct engulfment, projection, or septation of lysosome membranes [10]. Macro- and microautophagy are both capable of engulfing large cellular components through selective and nonselective mechanisms. CMA is an extensive form of autophagy found in almost all cells and tissue types in higher eukaryotes and involves the direct recognition of targeted proteins containing the KFERQ motif, which is recognized by chaperone proteins, such as the heat shock protein (Hsc-70). These proteins are then delivered directly to lysosomes via the lysosomal-associated membrane protein 2A (LAMP-2A) [11, 12]. Studies increasingly support the notion that dysregulation of the CMA pathway plays a role in multiple NDs including Alzheimer's Disease (AD), Parkinson's Disease (PD), Amyotrophic Lateral Sclerosis (ALS), and Frontotemporal Lobar Degeneration [12].

Neurons are of the postmitotic cell type and do not go through cytokinesis, and thus, cell division cannot reduce unnecessary deposited proteins. Therefore, the primary cause of NDs is pathological protein accumulation within neuronal cells, and this could be prevented by autophagic degradation. Hence, autophagy activation plays an important role in the treatment of NDs and provides a strategic platform for disease management [13, 14]. In this review, we discussed the beneficial effects of medicinal plants on NDs by targeting autophagy. Before describing the roles of herbal products in NDs, we provide a brief overview of autophagic processes at a molecular level and their roles in various NDs.

## 2. Molecular Process of Autophagy

Autophagy is highly inducible by starvation and other stress-related responses, and its activation causes a rapid increase in numbers of autophagosomes, which are generated on or near the endoplasmic reticulum [15]. In addition, membranes acquired from the Golgi complex, mitochondria, and plasma membrane also contribute to autophagosome formation [15]. Autophagosome establishment involves a multicomponent complex process, which is governed by multiple Atg proteins and related proteins. Of the 35 Atg proteins known, Atg1–10, 12–14, 16, and 18 are the “core Atg proteins” in yeast [16], and these proteins with Atg17, 29, and 31 participate in autophagosome formation. Other autophagic pathways such as pexophagy (autophagic peroxisome degradation) and cytoplasm-to-vacuole targeting share core Atg proteins [17, 18]. Furthermore, core Atg proteins are highly conserved in eukaryotes, including mammals, and function in a similar hierarchical manner in yeast [19].

Studies have shown that mTOR is essential for promoting adult stem cell differentiation, progenitor cell growth, and proliferation and has a great impact on the multipotent stem cell population [20, 21]. Interruption or deregulation of the autophagy system has been implicated in neurodegenerative issues such as AD, and A*β* assumes a significant role in this autophagy framework. Autophagy plays a substantial role in the generation and digestion of A*β* and the accumulation of tau. Much of the time, autophagy is directed by the phosphatidylinositol 3-phosphate kinase/AKT/mTOR/p70 ribosomal protein S6 kinase signaling pathway [22].

Various mTOR-dependent and mTOR-independent autophagy modulators have been found to have beneficial effects in the management of AD [23]. The mTOR pathway appears to be an adaptable player, and its modulation can affect both neuroprotective and cognitive processes. Although direct targeting of mTOR does not appear to have therapeutic potential, its indirect modulation by other signaling pathways is encouraging in this respect [24]. Targeting of rapamycin complex 1 (mTORC1) promotes cell growth in nutrient-rich environments by inducing the biosynthesis of proteins, lipid, and nucleotides and inhibiting cellular catabolism by suppressing the autophagic pathway [25, 26]. Aberrant mTOR signaling is associated with brain abnormalities and NDs. Even subtle defects in the mTOR pathway may have severe effects, including neurological and psychiatric disorders. Conversely, mTOR inhibitors can be useful in several neuropsychiatric alterations such as in brain cancer, brain ischemia, schizophrenia, autism, and NDs. mTOR has been linked to synaptic plasticity and the activation of autophagy [27]. Under nutrient-rich conditions, by direct association with the ULK1-Atg13-FIP200 complex, mTOR suppresses autophagy and mediates the phosphorylation-dependent inhibitions of the autophagy-related kinase activities of -13 (Atg13) and Unc-51-like kinase 1 (ULK1). Furthermore, the mTOR-mediated phosphorylations of Atg13 and ULK1 are inhibited under starvation conditions or after treatment with rapamycin. Rapamycin inhibits mTOR, and this triggers autophagy by dephosphorylating ULK1, ULK2, and Atg13 and activating ULK to phosphorylate FIP200 (FAK family-interacting protein of 200 kDa) [28, 29]. The existence of mTOR-independent regulation of mammalian autophagy resulted from the observation that intracellular inositol and inositol 1,4,5-trisphosphate (IP3) levels negatively regulate autophagy [30]. Furthermore, inhibition of inositol monophosphatase decreased levels of free inositol and IP3, which led to autophagy upregulation [31].

In postmitotic neuronal cells, basal autophagy movement is important, possibly because of their inability to dilute noxious components via cell division [32]. Autophagic activity is enhanced by various burdens, for example, by supplement starvation, hypoxia, or inflammation [33]. During certain physiological processes and pathological conditions, increased autophagy leads to cell death, the elimination of microorganisms entering cells, and cancer suppression [34]. Then again, the reduced autophagic potential is related to aging [35]. During autophagy, proteins are degraded into amino acids and, thus, provide a source of energy and raw materials for protein synthesis [36]. Hence, the dysregulation of autophagy may result in the aggregation of intracellular proteins. Furthermore, different types of autophagy dysfunctions can lead to ND or ND-like symptoms, such as the inhibition of autophagosome-lysosome fusion [37], reduced lysosomal acidification [38], or intracellular protein deposition [39].

**Figure 1:**
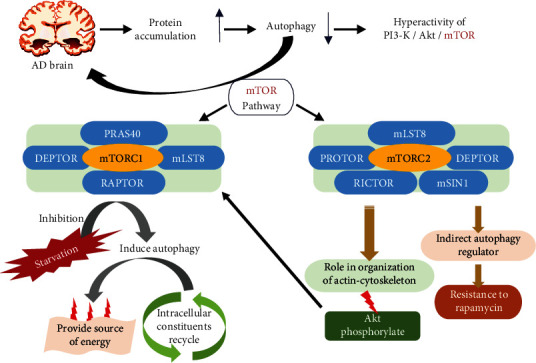
Molecular pathways in autophagy.

Two complexes, that is, mTORC1 and mTORC2, are responsible for the regulation of autophagy. mTORC1 consists of four different protein factors, viz., raptor (regulatory-associated protein of mTOR), deptor (DEP-domain containing mTOR-interacting protein), PRAS40 (proline-rich Akt substrate of 40 kDa), and mLST8 (mammalian lethal with SEC13 protein 8), whereas mTORC2 consists of rictor (rapamycin insensitive companion of mTOR), protor (protein observed with rictor), and mSIN1 (mammalian stress-activated mitogen-activated protein kinase-interacting protein) along with mLST8 and deptor [40, 41]. Starvation results in the activation of the mTORC1 complex, which stimulates autophagy resulting in the recycling of intracellular components as a source of energy [42]. In addition, the phosphorylation of Akt by the mTORC2 complex results in the activation of the mTORC1 complex **(**Figure 1**)**.

## 3. Autophagy, Immune Response, and Neurodegeneration

Most cellular stress-response pathways, including those that regulate immunological responses and inflammation, interact with the autophagy machinery [43–45]. The autophagy pathway/proteins have a complex reciprocal relationship with immunity and inflammation; autophagy proteins are involved in both the stimulation and suppression of immune and inflammatory responses, and immune and inflammatory signals are involved in both the stimulation and suppression of autophagy [44].

Autophagic interference with type I interferon responses occurs either directly by targeting signaling molecules within the pathway, beginning with RIG-I-like receptors or cGAMP synthase (a cytosolic DNA sensor) and progressing to the stimulator of the interferon gene (STING) and interferon regulatory factors, or indirectly by removing agonist sources that activate these pathways [46–49]. The p62 receptor appears to have a function in preventing T-cell receptor- (TCR-) mediated NF-*κ*B signaling via Bcl10. Although p62 initiates signaling, it also functions as a receptor to degrade Bcl10, which becomes ubiquitinated in response to TCR activation. Therefore, this strategy may protect cells against NF-*κ*B hyperactivation as a result of TCR signaling [50].

Using fly genetics, researchers show that deregulation of cyclin-dependent kinase 5 (Cdk5) activity disrupts autophagy, leads to an overactive innate immune response, and results in dopamine neurodegeneration in Drosophila [51]. It was demonstrated that an overactive innate immune response was sufficient to trigger neuronal cell death. Intriguingly, inhibiting the NF-*κ*B transcription factor in neurons lowers neuronal loss and downregulates the innate immune response genes in the Cdk5-deficient background [52].

Many NDs are linked with inflammatory responses in glia, which may contribute to pathology, and autophagy in glial cells may play a role in regulating these processes [53]. Microglia, as a key immune cell in the brain, influences phagocytosis and inflammation in age-related NDs [54]. When the LC3B and Atg7 genes were inhibited, microglia failed to degrade extracellular A*β*, indicating that autophagy function impairment in microglia may contribute to CNS-degenerative neurological disease [55]. Astrocytes are specialized glial cells in the brain and spinal cord and have been linked to the development of various NDs such as AD, PD, and ALS [56, 57]. Trifluoperazine-induced autophagy was implicated in astrocyte protection against bilirubin-induced cytotoxicity [58]. Recently, it has been demonstrated that Atg5 knockdown reduced astrocyte development in vivo, but Atg5 overexpression resulted in excessive astrocyte differentiation in vivo [59].

## 4. Autophagy and Neurodegenerative Disorders

### 4.1. Autophagy in Alzheimer's Disease (AD)

AD is characterized by the depositions of A*β* and tau in the brain. Under normal conditions, the production and clearance rates of A*β* are balanced, and A*β* is not deposited inside neuronal cells. Enhanced aggregation of A*β* peptides has been found in AD patients, and it is well recognized that failure of the autophagic system is a characteristic of AD. Recently, it was shown that autophagy enhanced the protein degradations of A*β* and tau [60]. During autophagy, autophagosomes enclosing A*β* facilitate its degradation by fusing with lysosomes. In addition, the microglial inflammatory response is regulated by autophagy, and dysregulation of autophagy damages neurons by exacerbating NLRP3 inflammasome signaling [55].

The C-terminal fragments of the amyloid precursor protein (APP) might be an etiological trigger for AD [61]. The cleavage of APP by BACE-1 produces C99 fragments. Reductions in autophagy (inhibition of autophagosome production or prevention of autophagosome fusion with lysosomes) result in increased C99 levels [62, 63]. Conversely, enhanced autophagy, either by mTOR suppression or by starvation, enhances C99 clearance in degenerative lysosomes [62, 64]. Also, lysosomes are disrupted by phagocytosis of the A*β* peptide, which results in the release of a lysosomal proteolytic enzyme (cathepsin B), which, in turn, stimulates pyrin domain containing 3 inflammasomes and leads to the production of proinflammatory and neurotoxic factors via the interleukin 1 beta pathway [65]. Stimulation of the autophagic system via cystatin B deletion decreases A*β* aggregation and has protective effects in mouse models of AD [66]. Tau protein stabilizes the microtubule, but its hyperphosphorylation reduces its affinity for microtubules and results in microtubule entanglement. Thus, the elimination of the tau protein by the autophagic system is required to address NDs [67, 68]. In addition, enhanced accumulation of the tau protein in the presence of the autophagic inhibitor (3-methyladenine) suggests that autophagy is required to prevent tau aggregation [69].

Various approaches used to upregulate the autophagic system have potential use for the management of AD [70]. Rapamycin, an inhibitor of the mTOR pathway, decreased A*β* deposition and prevented AD development by enhancing autophagy in an animal model of AD [71, 72], and the multifunctional protein p62 has been linked to neuropathological inclusions in various NDs and with the degradations of A*β* and tau. The ubiquitin-binding domain and the LC3- (microtubule-associated protein 1 light chain 3-) interacting regions of p62 are two functional domains. Enhancing brain p62 expression promoted autophagy and led to cognitive improvement in an animal model of AD, whereas removing the LC3-interacting region domain disrupted A*β* clearance by preventing autophagy [73]. In another study, latrepirdine was found to decrease A*β* aggregation by stimulating the Atg5-dependent autophagy in an animal model [70]. These reports indicate that autophagy is disrupted in AD and that regulating the autophagic system offers a reasonable therapeutic approach.

### 4.2. Autophagy in Parkinson's Disease (PD)

Autophagy and the ubiquitin-mediated pathway eliminate misfolded proteins in healthy cells, but both of these pathways are disrupted in PD, which results in the aggregation of misfolded proteins [74]. One of the important hallmarks of PD is the deposition of misfolded *α*-synuclein into intraneuronal inclusions known as Lewy bodies (LBs). *α*-Synuclein is susceptible to degradation by chaperone-mediated autophagy [75]. In familial PD, lysosomes are unable to engulf the mutant *α*-synuclein because of its high affinity for the lysosomal receptor (LAMP-2A), which in turn prevents *α*-synuclein degradation by shielding the substrate from CMA [76]. Furthermore, autophagosome and dysfunctional lysosome accumulations were found in postmortem PD brain samples [77], which highlighted the pathogenic role of autophagy in PD. Enhanced *α*-synuclein levels have been found in the lysosomal dysfunction, indicating a close link between autophagy and *α*-synuclein degradation. Various studies have reported that autophagy can degrade all forms of *α*-synuclein [77, 78] and that proteasomes also degrades monomeric *α*-synuclein [79].

Overexpression of *α*-synuclein is caused by mutations in *SNCA*, which encodes for *α*-synuclein, and these are sufficient to cause the progression of PD. Excess *α*-synuclein levels damage the autophagy system by hindering small GTPase Rab-1A [80]. Autophagy contributes in various ways to the protection of neural cells, but *α*-synuclein accumulation enhances protein aggregation levels, counters the effective clearance of misfolded protein, and induces neuronal cell apoptosis [80]. Moreover, *α*-synuclein mutation has been suggested to impair CMA [76, 81]. These results indicate that the regulations of more than one type of autophagy by *α*-synuclein mutations have toxic effects on neuronal cells.

### 4.3. Autophagy in Amyotrophic Lateral Sclerosis (ALS)

ALS is a paralytic condition defined as motor neuronal dysfunctions in the brain and spinal cord resulting in muscle atrophy. Mutations in TAR DNA-binding protein and superoxide dismutase 1 (SOD1) are common causes of familial ALS [82], and it has been established that autophagy is linked with ALS. Rat LC3 is vital for autophagy, and the formation of LC3-II from LC3-I has been suggested to provide a simple means of controlling autophagy. LC3-II overexpression has been reported in mutant SOD1G93A transgenic mice. In addition, enhanced autophagosomes were strongly associated with reduced mTOR phosphorylation in various genetic ALS models [83]. A growing number of studies have established that mutations in autophagy-associated proteins are well correlated with the pathogenesis of ALS. The endosomal sorting complexes required for transport (ESCRTs) are responsible for sorting transmembrane proteins into the inner vesicles of the multivesicular body (MVB) during endocytosis. Reductions in ESCRT subunits inhibit either autophagosome-MVB fusion or amphisome-lysosome fusion and are considered to be linked with ALS [84]. Ubiquilin-2 (a proteasome shuttle factor) has an important role in the generation of autophagosomes. Mice with mutated *UBQLN2* exhibit neuronal loss, cognitive deficits, and short lifespans [85].

## 5. Beneficial Effects of Medicinal Plants on NDs by Targeting Autophagy

**Figure 2:**
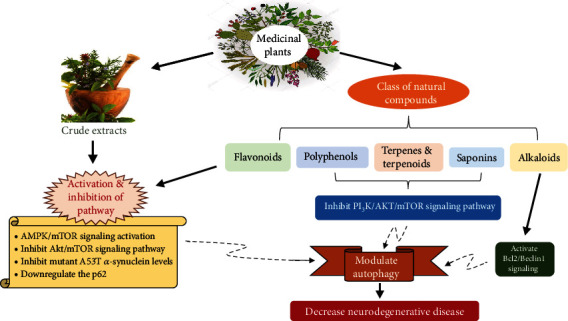
Different classes of natural compounds that modulate autophagy and suppress neurodegeneration by activating or inhibiting different molecular pathways.

Medicinal herbs have become increasingly important in the quest for more effective and adjunctive treatments [86–89]. Various pharmacological studies have reported that the active components of herbal medicines show therapeutic benefits in NDs via different mechanisms such as by increasing neurogenic activity, inhibiting cholinesterase activity, and controlling A*β*, tau, and *α*-synuclein metabolism by targeting autophagy [90–92].  Table 1 provides a summary of classes of natural compounds that reduce neurodegeneration by regulating autophagy **(**Figure 2**)**.

### 5.1. Crude Extracts

*Radix Polygalae* extract was reported to decrease A*β* and mutant A53T *α*-synuclein levels by activating AMPK/mTOR signaling to stimulate autophagy in Chinese hamster ovary cells (transfected with APP and BACE1) and PC-12 cells, respectively [93, 94]. *Withania somnifera* extract had a protective effect in ALS by downregulating p62 (a classical autophagy receptor), thereby promoting autophagy in the motor neurons of SOD1^G93A^ mice [95]. In another study, *Ginkgo biloba* extract repressed microglial inflammation and enhanced cognitive functions by regulating the mechanism moderately involved in the activation of autophagy [96].

### 5.2. Saponins

Ginsenoside-Rg2, a bioactive compound obtained from *Panax ginseng* induces autophagy in an AMPK/ULK1-dependent manner. Rg2 increased the clearance of aggregated proteins and enhanced cognitive function by inducing autophagy in an AD mouse model [97]. The protopanaxadiol derivative DDPU (1-(3,4-dimethoxyphenethyl)-3-(3-dehydroxyl-20(s)-protopanaxadiol-3*β*-yl)-urea) increased A*β* clearance by inducing autophagy via the PI_3_K/AKT/mTOR signaling pathway by inhibiting PI_3_K and decreased A*β* generation by restraining PERK/eIF2*α* signaling-mediated BACE1 translation [98]. In addition, *Radix Polygalae* derived onjisaponin B enhanced mutant *α*-synuclein degradation by autophagy induction by activating the AMPK/mTOR signaling pathway [93].

### 5.3. Alkaloids

Alkaloids are the important active components in herbal medicines and exert beneficial effects on NDs by inducing autophagy and inhibiting cholinesterase activity [99, 100]. Alkaloids isolated from *Dendrobium nobile* enhanced autophagic flux via autophagosome generation and stimulated Beclin-1 expression [101], and berberine has been reported to stimulate autophagy by activating Bcl2/Beclin-1 signaling, thus increasing A*β* clearance, and to improve cognitive functions in a mouse model of AD [102]. Furthermore, it has been reported that berberine can bypass the blood-brain barrier [103]. TDP-43 (43 kDa nuclear protein TAR DNA-binding protein) is the main component of ubiquitinated inclusions in aggregated proteins in ALS [104, 105]. Berberine has therapeutic potential in ALS as it reverses TDP-43 proteinopathy by disrupting mTOR/p70S6K signaling and stimulating the autophagic degradation pathway [106]. Corynoxine isolated from *Uncaria rhynchophylla* is an established inducer of autophagy and enhances autophagosome generation and the elimination of *α*-synuclein in PC12 cells [107]. Isorhynchophylline, a main tetracyclic oxindole alkaloid obtained from *U. rhynchophylla*, has been used to manage NDs in East Asia for centuries. This alkaloid induces the Beclin-1-dependent autophagy-lysosome pathway and enhances the clearance of *α*-synuclein monomers and *α*-synuclein/synphilin-1 aggresomes from neuronal cells [108]. *Angelica sinensis*-derived n-butylidenephthalide enhanced motor functions in SOD1-ALS mice. The autophagy pathway was involved in the therapeutic mechanism, as n-butylidenephthalide treatment reduced LC3-II expression and increased mTOR levels [109]. In addition, conophylline from *Ervatamia microphylla* induced autophagy in Huntington disease and PD models [100, 110].

### 5.4. Flavonoids

Studies have established that flavonoids influence the autophagy system in some disorders [111, 112]. Silibinin isolated from *Silybum marianum* reduced neuronal damage via the BDNF/TrkB pathway by decreasing autophagy in the hippocampus [113]. In another study, wogonin enhanced autophagy by inhibiting the Akt/mTOR pathway and increasing A*β* clearance [114]. Hesperetin recovered A*β* damage-induced glucose utilization by downregulating A*β*-stimulated autophagy [115], and kaempferol has been reported to enhance autophagy and decrease ROS, apoptosis, and mitochondrial dysfunction in rotenone-exposed SH-SY5Y cells [116].

### 5.5. Polyphenols

Curcumin inhibits A*β* aggregation and ameliorates cognitive functions. The mechanisms responsible involve the stimulation of autophagy by downregulating the PI_3_K/Akt/mTOR pathway [117]. In amyloid-treated HT-22 cells, curcumin protected hippocampal neurons by inhibiting the abnormal formation of Beclin-1 and autophagosomes [118]. In an *in vitro* dopaminergic neuron model of PD, curcumin was involved in the modulation of autophagy and the clearing of *α*-synuclein aggregates [119]. Resveratrol is attracting attention because of its curative potential in AD and has been reported to reduce A*β* generation and restrain the development of AD by inhibiting apoptosis and regulating mitophagy [120]. Curcumin decreased the accumulation of A53T *α*-synuclein protein (related to early-onset PD) by downregulating mTOR/p70 ribosomal protein S6 kinase signaling and induced macroautophagy in SH-SY5Y cells [121]. In addition, resveratrol has been reported to protect against neural damage by activating mitophagy [122] and to stimulate autophagy and lysosomal degradation by regulating the AMPK/mTOR signaling pathway and reducing A*β* synthesis in HEK293 and N2a cells [123]. In mice, orally administered resveratrol crossed the blood-brain barrier, stimulated brain AMPK, and decreased A*β* deposition in the cerebral cortex [123]. The active component (2,3,5,4′-tetrahydroxystilbene-2-O-glycoside) in *Radix Polygoni Multiflori* was reported to hinder autophagy by decreasing Beclin-1 levels, thus enhancing cognitive function [124], and carnosic acid stimulated autophagy by activating the AMPK/mTOR pathway and inhibited A*β* deposition [125].

Resveratrol was observed to protect SH-SY5Y cells from rotenone-stimulated apoptosis and to increase *α*-synuclein degradation in *α*-synuclein-expressing PC12 cell lines by inducing autophagy. The mechanism of *α*-synuclein degradation in a cellular model of PD involved the regulation of mammalian SIRT1 (silent information regulator 2)/AMPK (AMP-activated protein kinase), which diminished LC3-II protein levels and increased *α*-synuclein clearance [126]. Resveratrol improved mitochondrial oxidative function by regulating the AMPK and SIRT1 pathways and increased macroautophagic flux by activating an LC3-independent pathway in early-onset PD fibroblasts [127]. In another study, resveratrol stimulated heme oxygenase-1 expression and inhibited dopaminergic cell death by controlling autophagic flux and, as a result, protected against rotenone-induced neuronal apoptosis in a PD model [128]. *Corema album* polyphenol fractions promoted nontoxic *α*-synuclein formation and, thus, reduced its toxicity and aggregation in cells by enhancing autophagic flux and reducing oxidative stress [129]. In addition, *Arctium lappa*-derived arctigenin inhibited the generation and enhanced the clearance of A*β* by inducing autophagy by inhibiting AKT/mTOR signaling and AMPK/Raptor pathway activation in an animal model of AD [130].

### 5.6. Terpenes and Terpenoids

Recently, monoterpenes have been identified to be autophagy modulators [131]. Cubeben, a *Piper cubeba* sesquiterpene, decreased A*β* toxicity in primary neuronal cells, recovered autophagy via PI3K/AMPK signaling, and suppressed the inhibition of mTOR [132]. In a PD model, geraniol (an acyclic monoterpene) protected neurons against rotenone stress by restoring mitochondria, reducing *α*-synuclein levels, and increasing autophagic flux [133]. Cucurbitacin E (a terpenoid phytosterol) partially protected PC12 neurons from PD simulating toxins, significantly decreased Beclin-1 autophagy, increased autophagosome activities, and eliminated toxic deposits [134].

## 6. Nanomaterials, Autophagy, and NDs

The need for innovative therapeutic approaches for NDs, as well as the limits imposed by the BBB, is driving the use of nanotechnology in the delivery of targeted drugs to the CNS. Because of their physical and chemical properties, nanomaterials can be excellent drug carriers to the brain [135, 136]. Nanoparticles (NPs) stimulated intracellular autophagy, enhanced autophagosome breakdown, increased A*β* clearance in brain cell cultures, and decreased A*β*-stimulated cytotoxicity [137]. The use of nanocarriers that encapsulate molecules may improve drug transport through the BBB in NDs and target key brain areas for regenerative processes [135]. Quercetin is a natural antioxidant that has a low capacity to cross the BBB and is easily eliminated. Recently, it has been demonstrated that quercetin-modified gold-palladium NPs increase the clearance of intracellular A*β* via autophagy activation and, thereby, decrease A*β*-induced neurotoxicity [138]. This study paves the way for NPs to encapsulate natural products capable of modulating autophagy in the management of various NDs.

## 7. Conclusion and Future Prospects

Autophagy is an important process under normal and pathologic conditions. Studies have shown that the dysregulation of autophagy is involved in the pathogeneses of neurological disorders and suggested possible neuroprotective strategies to mitigate neurological disorders by managing the autophagy system. Several bioactive compounds derived from medicinal plants are believed to have the capabilities to control autophagy and treat NDs by targeting autophagic pathways. Regulation of the autophagic pathway is now viewed as an exciting drug developmental strategy because it is believed that the targeted control of autophagy offers a means of managing NDs.

## Figures and Tables

**Table 1:** Natural compounds that inhibit neurodegeneration via autophagy.

Natural sources	Signaling	Effects	References
*Crude extracts*			
*Radix Polygalae*	AMPK/mTOR	Decrease A*β* and mutant A53T *α*-synuclein levels	[93, 94]
*Withania somnifera*	Downregulate the p62 (a classical autophagy receptor)	Promote autophagy in motor neuron	[95]
*Saponins*			
Ginsenoside-Rg2	AMPK-ULK1-dependent and MTOR-independent	Aggregated protein clearance and enhanced cognitive function	[97]
DDPU	PI3K/AKT/mTOR and PERK/eIF2*α*	Clearance of A*β* and decreased A*β* generation	[98]
Onjisaponin B	AMPK/mTOR	Enhances mutant *α*-synuclein degradation	[93]
*Alkaloids*			
Berberine	Bcl2/Beclin-1	Clearance of A*β* and improves cognitive function	[102]
Isorhynchophylline	Beclin-1	Clearance of *α*-synuclein monomers and *α*-synuclein/synphilin-1 aggresomes	[108]
n-Butylidenephthalide	mTOR	Enhanced motor functions	[109]
*Flavonoids*			
Silibinin	BDNF/TrkB	Reduces neuronal damage	[113]
Wogonin	Akt/mTOR	Clearance of A*β*	[114]
Hesperetin	IRS-PI3K-Akt	Recovers A*β*-damage glucose utilization	[115]
*Polyphenols*			
Curcumin	PI3K/Akt/mTOR and mTOR/p70 ribosomal protein S6 kinase	Inhibits A*β* aggregation, improves cognitive function and decreased A53T *α*-synuclein accumulation	[117, 118]
Resveratrol	AMPK/mTOR	Decreased A*β* synthesis	[123]
2,3,5,4′-tetrahydroxystilbene-2-O-glycoside	Beclin-1	Cognitive function	[124]
Carnosic acid	AMPK/mTOR	Inhibits A*β* deposition	[125]
Arctigenin	AKT/mTOR	Enhanced A*β* clearance	[130]
*Terpenes*			
Cubeben	PI3K/AMPK/mTOR	Decreased A*β* toxicity	[132]
Geraniol	Increased Atg5-7-12	Reduce *α*-synuclein	[133]
Cucurbitacin E	Regulate autophagy lysosomal pathway	Eliminate toxic deposits	[134]
